# The association between an energy-adjusted dietary inflammatory index and inflammation in rural and urban Black South Africans

**DOI:** 10.1017/S136898002100505X

**Published:** 2022-12

**Authors:** Maylene Ferreira, H Toinét Cronjé, Tertia van Zyl, Nicola Bondonno, Marlien Pieters

**Affiliations:** 1 Centre of Excellence for Nutrition, North-West University, Potchefstroom X6001, South Africa; 2 Medical Research Council Unit for Hypertension and Cardiovascular Disease, North-West University, Potchefstroom, South Africa; 3 Section of Epidemiology, Department of Public Health, University of Copenhagen, Copenhagen, Denmark; 4 Institute for Nutrition Research, School of Medical and Health Sciences, Edith Cowan University, Perth, Australia; 5 The Danish Cancer Society Research Centre, Copenhagen, Denmark

**Keywords:** Urbanisation, Non-communicable disease risk, African diet, Dietary inflammatory potential, Alcohol

## Abstract

**Objective:**

To quantify the inflammatory potential of the diet of rural and urban Black South Africans using an adapted energy-adjusted dietary inflammatory index (AE-DII) and to investigate its relationship with inflammatory and cardio-metabolic disease risk markers. Dietary inflammatory potential has not been investigated in African populations.

**Design:**

Cross-sectional investigation.

**Setting:**

Rural and urban sites in the North West province of South Africa.

**Participants:**

1885 randomly selected, apparently healthy Black South Africans older than 30 years.

**Results:**

AE-DII scores ranged from –3·71 to +5·08 with a mean of +0·37. AE-DII scores were significantly higher in men (0·47 ± 1·19) than in women (0·32 ± 1·29), and in rural (0·55 ± 1·29) than urban participants (0·21 ± 1·19). Apart from its dietary constituents, AE-DII scores are primarily associated with age, rural–urban status and education. Contrary to the literature, alcohol consumption was positively associated with AE-DII scores. Of the four tested inflammatory and thirteen cardio-metabolic biomarkers, the AE-DII was only significantly negatively associated with albumin and HDL cholesterol, and positively with waist circumference and fasting glucose, upon full adjustment.

**Conclusion:**

Rural men consumed the most pro-inflammatory diet, and urban women the least pro-inflammatory diet. The diet of the participants was not overtly pro- or anti-inflammatory and was not associated with measured inflammatory markers. The inflammatory potential of alcohol at different levels of intake requires further research. Understanding dietary inflammatory potential in the context of food insecurity, unhealthy lifestyle practices and lack of dietary variety remains limited.

Chronic inflammation is a central component of all the leading non-communicable causes of mortality globally, including CVD, diabetes, chronic obstructive pulmonary disease, cancer and dementia^(1,2)^. Current evidence suggests that the alleviation of a chronic inflammatory response could delay the progression of non-communicable diseases (NCD)^(3)^ and reduce all-cause mortality risk^(4)^. One intervention strategy towards reducing inflammation is to lower the inflammatory potential of diets.

The role of diet as a modulator of inflammation in the context of NCD has been thoroughly described. For example, anti-inflammatory diets characterised by high intakes of a variety of fruit and vegetables, plant-based protein sources and unsaturated fats have been associated with reduced risk of chronic inflammation and mortality^(5,6)^. On the other hand, pro-inflammatory diets characterised by low fibre, high sugar and high saturated fat have been associated with increased levels of pro-inflammatory cytokines and chronic inflammation^(7)^.

Indices such as the Dietary inflammatory index (DII^®^) were developed to quantify the inflammatory potential of whole diets in an attempt to move away from focusing on intakes of individual pro- or anti-inflammatory nutrients in isolation. The DII^®^ has been updated twice since it was introduced in 2009^(8)^, based on updated literature^(9)^ and then taking total energy intake into account in an energy-adjusted version of the DII^®^ (E-DII^(10)^). Since its development, the DII^®^ has been associated with numerous plasma inflammatory markers and several health outcomes^(11)^.

Although these indices have been successfully used to determine the relationship between an individual’s diet and several health outcomes, less is known regarding the dietary inflammatory potential of African population groups whose dietary patterns and intakes differ from previous studies that have applied the DII^®^. Traditional African diets, which consist mainly of a monotonous dietary pattern of indigenous foods, are different from both Western and Mediterranean diets (for which most of the evidence exist) and need to be explored in relation to their inflammatory characteristics.

Within the context of South Africa, for example, the consumption of traditional African diets is varied, with more traditional intakes found in rural environments with a transition into Westernised dietary patterns observed in urban populations. Participants in the current investigation formed part of the Prospective Urban and Rural Epidemiology (PURE) study^(12)^, which specifically recruited and compared rural- and urban-living Black South Africans. Dietary intake in these groups was typically informed by their socio-economic status^(13)^. Overall, rural participants followed a low energy, low fat diet whereas urban participants followed a more Westernised high energy, high-fat diet with marked increases in the variety of foods consumed^(14)^.

Understanding the functioning of widely used metrics, such as the DII^®^, is of particular importance in low- and middle-income countries where 85 % of the global NCD mortality burden is carried^(15)^. The ‘nutritional transition’ that accompanies the urbanisation of low- and middle-income countries is often referred to as a key player in the increasing prevalence of NCD^(16)^ in these regions. Understanding the applicability of the DII^®^ in these populations is, therefore, crucial to investigating whether this increase in NCD can be explained by an increase in the inflammatory potential of the diet with urbanisation.

This article describes the application of an adapted E-DII to a South African context. We aim to describe the inflammatory potential of the diet of rural- and urban-living Black South Africans using an adapted version (AE-DII) of the E-DII^(9,10)^. We hypothesised that the differences in the lifestyle and dietary patterns between these two groups would be reflected in their dietary inflammatory scores. In addition, we hypothesised that the AE-DII would be associated with several inflammatory and cardio-metabolic disease risk markers.

## Methods

### Study population

This is a cross-sectional investigation of 2010 Black South African adults residing in distinct rural or urban sites within the North-West Province (NWP) of South Africa (SA). These individuals took part in the baseline data collection of the PURE-NWP-SA study^(12,13)^. Participants were eligible to participate if they were apparently healthy (not suffering from any acute or previously diagnosed chronic illness) and older than 30 years, at the time of study enrolment. With the exception of the availability of dietary recall data, no additional criteria were applied to inclusion in the current investigation. Participants with self-reported energy intake of more than 30 000 kJ (over-reporters) or fewer than 3000 kJ (under-reporters) were excluded from the analysis, resulting in a final cohort size of 1885^(17)^.

### Data collection

Data on socio-economic status, lifestyle (including smoking and alcohol consumption) and medication use were gathered using validated questionnaires filled out by participants under the guidance of qualified field workers. BMI was calculated from weight (measured to the nearest 0·01 kg) divided by height (as meters, measured to the nearest 0·1 cm) squared (kg/m^2^). The Omron HEM-757 (Omron Healthcare) was used to determine brachial systolic and diastolic blood pressure and heart rate. These measures were taken in the supine position after 5 min of rest, with the second of two measures recorded. Waist circumference (WC) was measured in cm using a Holtain unstretchable metal tape.

### Dietary assessment

Trained field workers captured data regarding food consumption and dietary habits within the previous month using a validated quantitative FFQ specifically developed and tested for this population^(18)^. Recall tools such as a culturally sensitive food-portion photograph book, utensils and plastic food replicates were also used^(19)^. Food and nutrient intake data were coded according to the Medical Research Council’s Food Composition Tables (FCT)^(20)^ and the amounts determined in grams, using the Medical Research Council Food Quantities Manual^(21)^. Household measures of food were converted to grams using standard tables^(21)^. Food intake and nutrient analyses were performed by the South African Medical Research Council at their Biostatistics Unit^(20)^. The FCT are continuously updated to ensure accuracy and appropriate nutrient coverage^(20)^.

### AE-DII

The AE-DII, adapted for the PURE-NWP-SA participants, included thirty-seven of the forty-five suggested food parameters^(9)^. The following food components were excluded because they were not captured in the FCT or they were not consumed by the PURE-NWP-SA participants: eugenol, garlic, ginger, oregano, pepper, rosemary, saffron and turmeric. In accordance with prior investigations, two additional adaptions were made to the AE-DII calculation performed for this investigation^(22)^. Total fat was excluded, as the inclusion of individual fat components (PUFA and MUFA, trans- and saturated fat and cholesterol) collectively compensates for the contribution of total fat. Alcohol consumption data of participants who consumed more than 40 g of alcohol per day were excluded because of the prior evidence that consumption exceeding 40 g/d no longer exerts any anti-inflammatory effects^(22,23)^.

The AE-DII was calculated in accordance with the published procedures^(9,10)^. First, individual daily intake of each food/nutrient was standardised to represent a 1000 kcal (4180 kJ) total energy intake. Second, a z-score was computed by subtracting the cohort mean from each individual’s food/nutrient parameter and then dividing it by the cohort sd. To reduce the effect of right skewing, z-scores were expressed as cumulative proportions, and subsequently multiplied by two with one subtracted from the product. Resulting values for each food parameter were then multiplied by its parameter-specific inflammatory effect score, and summed to deliver an overall AE-DII measure for each participant. AE-DII scores are positively correlated to inflammatory potential; the higher an individual’s score, the higher their dietary inflammatory potential while negative scores indicate consumption of an anti-inflammatory diet^(9)^. The AE-DII adapted for the PURE-NWP-SA cohort spanned a theoretical range of –7·65 to +6·67.

### Flavonoids

The following flavonoid subclasses were included in the calculation of the AE-DII: anthocyanidins, flavan-3-ol, flavanones, flavones, flavonols and isoflavones. Since the FCT does not include polyphenol content estimates, we determined the flavonoid content of the foods consumed by the PURE-NWP-SA participants, using the Phenol Explorer database^(23)^. Phenol Explorer provides the content of flavonoid compounds for raw food, expressed as mg/100 g fresh weight. Therefore, for all raw and fresh foods, the flavonoid quantity for each food item was used as is. To calculate the flavonoid content for cooked or dried foods, the moisture content of the same fresh food, provided by the FCT, was subtracted from 100 to provide the remaining solid content for each food. The quantity of each flavonoid compound was summed for their respective subclasses (anthocyanidins, flavan-3-ol, flavanones, flavones, flavonols and isoflavones) to provide the total amount of each subclass specified in the AE-DII, for each food item.

### Biochemical analysis

Fasting blood samples were collected from the antecubital vein between 07.00 and 11.00 and centrifuged at 2000 × *g* for 15 min within 30 min after collection and stored at –80°C until analysis. Plasma glucose (fluoride tubes) was quantified by a Vitros DT6011 Chemistry Analyser (Ortho-Clinical Diagnostics). Glycated Hb (HbA_1c_) was determined with the D-10 Hb testing system (Biorad, Hercules) from EDTA blood. Serum high-sensitivity C-reactive protein (hs-CRP), albumin, total cholesterol, HDL cholesterol (HDL-C), TAG, alanine transaminase (ALT), aspartate transaminase (AST), gamma-glutamyl transferase (GGT) and creatinine were quantified using the Konelab20i™ auto-analyser (Thermo Fischer Scientific). LDL cholesterol (LDL-C) was calculated using the Friedewald–Levy–Fredrickson formula^(24)^. Serum IL-6 was analysed through enzyme immunoassays (Elecsys 2010). Soluble urokinase-type plasminogen activator receptor was quantified using the suPARnostic^®^ ELISA (ViroGates).

### Statistical analyses

Statistical analyses were conducted using R 4.0.1^(25)^. Data distribution was evaluated through visual inspection of histograms. Variables are expressed as mean ± sd or median (interquartile range) based on their distribution. As most of the data were skewed, non-parametric testing was used where appropriate. Log transformations were applied to skewed variables prior to linear modelling when residuals were not normally distributed. Regression coefficients for log-linear models were transformed [(*e* ^coefficient^–1) × 100] and are reported as percentage change in outcome per unit change in predictor.

Mann–Whitney *U* tests were used to compare data between dichotomous sex, rural–urban status, smoking and alcohol consumption groups. Smoking and alcohol consumption status were dichotomised to *ever* and *never* users/consumers due to the small size of the *former* category represented in each. ANOVA and Tukey’s *post hoc* analysis were used when comparing differences in AE-DII predictors across AE-DII quartiles and aggregated sex rural–urban status groups. ANCOVA was used when comparing AE-DII quartiles and aggregated sex rural–urban status groups. Univariate linear regression models were performed to assess the associations of continuous AE-DII with various demographic and lifestyle predictors. Multivariable linear regression models were performed to determine the association between AE-DII and inflammatory and other cardio-metabolic risk markers. Primary models adjusted for conventional covariates including age, sex, rural–urban status, education, smoking status, as well as the use of aspirin or anti-inflammatory medication. Secondary models additionally adjusted for BMI (as a conventional covariate), alcohol consumption (g/d) (because it was capped at 40 g/d in the AE-DII calculation) and energy intake (which was normalised through the standardisation step) due to their associations with AE-DII scores in the PURE-NWP-SA cohort). Sensitivity analyses entailed removal of participants with potential acute inflammation, defined as CRP > 10 mg/l (*n* 525) and the use of an AE-DII score computed without a restriction on the contribution of alcohol consumption greater than 40 g/d. Reported results were robust to these sensitivity analyses, unless specified otherwise in the results text. A two-sided *P* < 0·05 denoted statistical significance in all analyses.

## Results

The AE-DIIs in the PURE-NWP-SA cohort ranged from –3·71 to +5·08, spanning 61 % of the theoretical PURE-NWP-SA AE-DII range (–7·67 to +6·67). The mean cohort AE-DII was 0·37 ± 1·25, which indicates an overall mean pro-inflammatory diet. Demographic characteristics of the study cohort overall and stratified by AE-DII quartiles are reported in Table 1. A summary of the intake of specific pro- and anti-inflammatory dietary components in the full cohort and across AE-DII quartiles is reported in Supplemental Table 2.

**Table 1:** Demographic characteristics of the PURE-NWP-SA cohort overall and stratified by AE-DII quartiles

			AE-DII quartiles							
	Full cohort		1 (–3·708; –0·467)		2 (–0·466; 0·296)		3 (0·297; 1·174)		4 (1·175; 5·080)	
Variable	Median/%*	IQR	Median/%*	IQR	Median/%*	IQR	Median/%*	IQR	Median/%*	IQR
*n*	1858		465		465		464		464	
Age (years)	48·0	42·0–57·0	50·0	43·0–58·0	48·0	42·0–57·0	48·0	41·0–55·0	47·0	41·0–56·0
Women (%)	63·0		69·0		60·0		62·0		60·0	
Rural (%)	48·0		42·0		40·0		48·0		61·0	
Education (%)										
No education	35·0		30·0		31·0		35·0		43·0	
1–12 years	64·0		69·0		69·0		63·0		56·0	
> 12 years	1·00		1·00		0·50		2·00		1·00	
Energy intake (kJ/d)	7422	5509–10 149	7161	5367–9760	7640	5871–10 293	7136	5313–9675	7581	5660–11 224
Alcohol consumption										
Status (% ever)	44·0		37·0		45·0		47·0		48·0	
Per day (g)†	16·8	6·93–35·6	15·4	5·71–25·9	15·4	5·93–28·6	15·4	5·71–22·3	26·9	10·8–65·1
Per day (g)‡	11·6	5·29–22·9	14·3	5·71–23·1	11·4	5·71–22·9	11·6	4·29–22·9	11·6	5·61–22·9
Smoker (% ever)	56·0		49·0		56·0		57·0		62·0	

PURE, Prospective Urban and Rural Epidemiology; NWP, North-West Province; SA, South Africa; AE-DII, adjusted energy-adjusted dietary inflammatory index; IQR, inter quartile range.

Data are presented as median (interquartile range) or frequency.

* Percentage of individuals in each category.

† Only alcohol consumers included.

‡ Only alcohol consumers included, alcohol intakes > 40 g/d are excluded in accordance with the manner in which alcohol consumption was used in the AE-DII calculation.

### Demographic and lifestyle predictors of the AE-DII

AE-DII scores were significantly higher in men (0·47 ± 1·19) than in women (0·32 ± 1·29, *P* = 0·01), and in rural (0·55 ± 1·29) than urban participants (0·21 ± 1·19, *P* < 0·001). This pattern is also observed in the higher proportion of male participants and rural dwellers across AE-DII categories when using univariate linear regression (*P* < 0·001). Age decreased across the quartiles, suggesting that older participants consumed a more anti-inflammatory diet. Average AE-DII scores decreased by 0·01 units per age year (*P* < 0·001). The percentage of educated participants decreased across the AE-DII quartiles. Although not evident across quartiles, a positive association was observed between daily energy consumption and continuously evaluated AE-DII scores (0·03-unit AE-DII higher per 1000 kJ, *P* = 0·01). Participants who had ever smoked or consumed alcohol had higher AE-DIIs (0·46 ± 1·24 and 0·40 ± 1·19) compared with those who had never smoked (0·26 ± 1·27, *P* = 0·001) or consumed alcohol (0·20 ± 1·29, *P* = 0·001). This association was also observed in the increased frequency of ever *v*. never users/consumers across quartiles (*P* < 0·001). When all predictor variables were simultaneously included in a regression model with continuous AE-DII as the outcome; age, rural–urban status, education, energy intake and alcohol consumption remained statistically significant predictors of AE-DII, while the contribution of sex and smoking status were attenuated. In this model, alcohol consumption was statistically the strongest single predictor of AE-DII, resulting in a 0·015 higher AE-DII per gram alcohol consumed. When alcohol consumption greater than 40 g/d was excluded (as done in the AE-DII score calculations), no across-quartile or linear associations between alcohol consumption and the AE-DII were observed.

Many of the non-nutrient predictors of the AE-DII were strongly influenced by rural–urban status and sex. A much higher proportion of rural (50 %) compared with urban (21 %) participants were uneducated, with both groups having more uneducated men than women. Conversely, urban dwellers, particularly men, were more likely to have smoked (58 %) or consumed alcohol (55 %) compared with their rural living (53 and 33 %) or female counterparts (see online supplementary material, Supplemental Table 2).

### Urbanicity as a predictor of the AE-DII

When considered as an aggregated sex/rural–urban demographic status (Table 2), rural men consumed the most pro-inflammatory diet, followed by rural women (0·75 ± 1·25 and 0·44 ± 1·30). Although urban women consumed the most anti-inflammatory diet, their mean AE-DII (0·19 ± 1·26) was still reflective of a pro-inflammatory diet. Urban men consumed the highest energy (considered pro-inflammatory in the original DII^®^), followed by urban women. Increased energy intake was also reflected in urban participants reporting higher consumption of almost all food components compared with their rural counterparts. Rural women consumed the lowest daily energy, also reflected in having the lowest consumption of nineteen of the thirty-seven food components investigated.

**Table 2:** Comparison of daily AE-DII food parameter consumption in men and women of rural or urban status

	Total population		Urban				Rural			
			Women		Men		Women		Men	
	Mean/median	sd/IQR	Mean/median	sd/IQR	Mean/median	sd/IQR	Mean/median	sd/IQR	Mean/median	sd/IQR
*n*	1585		578		390		585		305	
AE-DII	0·37	1·25	0·19	1·26	0·24	1·08	0·44	1·30	0·75	1·25
Pro-inflammatory										
Carbohydrate (g)	265	201–349	288	201–369	323	233–415	239	188–293	250	203–326
Cholesterol (mg)	157	87·5–265	224	147–330	225	159–344	102	59·7–157	108	63·6–157
Energy (kJ)*	7422	5509–10 149	8700	6269–11 528	9464	7212–12 386	6021	4925–7523	6786	5413–8594
Fe (mg)	13·8	6·38	14·3	6·69	16·8	7·65	11·6	4·36	13·1	5·52
Protein (g)	50·8	36·5–72·7	62·4	46·4–84·8	70·4	52·3–90·5	39·3	31·2–49·3	43·5	34·0–5·83
Saturated fat (g)	10·4	6·31–16·6	15·9	10·8–22·2	14·7	10·5–20·8	6·75	4·42–9·72	6·72	4·30–9·40
Trans fat (g)	0·20	0·07–0·56	0·52	0·26–0·97	0·43	0·20–0·78	0·08	0·04–0·17	0·10	0·04–0·18
Vitamin B_12_ (µg)	2·82	1·34–5·25	4·42	2·34–7·49	4·69	2·68–7·61	1·66	0·86–3·01	1·60	0·92–2·89
Anti-inflammatory										
Alcohol (g/d)	0·00	0·00–15·4	0·00	0·00–11·5	11·6	0·00–26·7	0·00	0·00–0·00	3·86	0·00–28·9
Alcohol (g/d)†	0·00	0·00–6·81	0·00	0·00–7·56	4·97	0·00–17·1	0·00	0·00–0·00	0·00	0·00–5·79
Anthocyanidins (mg)	0·13	0·00–0·43	0·43	0·00–1·86	0·21	0·00–0·61	0·11	0·00–0·21	0·05	0·00–0·16
*β*-Carotene (µg)	998	342–2069	1607	572–3027	1432	450–2445	856	230–1459	536	89·6–1090
Black tea/Rooibos (g)‡	1540	900–2100	1540	1100–3080	1540	900–2100	1800	900–2100	2100	1500–2100
Caffeine (g)§	840	308–1428	420	252–840	420	240–840	1428	816–1548	1428	420–1428
Fibre (g)	21·7	10·2	23·1	10·6	26·9	12·6	17·9	6·42	19·6	8·04
Flavan-3-ol (mg)	91·2	21·6–213	169	91·3–264	158	90·5–239	28·2	7·42–83·8	29·6	7·33–91·1
Flavones (mg)	2·82	0·77–6·39	3·04	0·68–6·69	2·60	0·45–6·42	2·73	1·23–5·51	3·13	1·02–6·21
Flavonols (mg)	21·0	6·16–39·8	35·1	20·3–50·7	32·5	21·0–46·0	6·47	2·31–18·2	6·65	3·52–21·6
Flavonones (mg)	0·80	0·15–8·06	4·50	0·52–19·6	2·89	0·58–15·0	0·15	0·05–1·31	0·34	0·10–1·77
Folic acid (µg)	369	267–491	370	259–511	458	324–635	332	253–422	371	262–464
Isoflavones (mg)	0·02	0·00–0·04	0·01	0·00–0·03	0·02	0·00–0·04	0·01	0·00–0·04	0·03	0·00–0·07
MUFA (g)	11·2	6·59–18·8	17·7	12·0–25·6	16·4	11·7–24·2	6·96	4·69–10·3	6·93	4·61–10·1
Magnesium (mg)	292	210–412	323	225–412	388	284–526	233	184–306	291	211–48
Niacin (mg)	13·1	9·37–18·9	15·5	10·9–21·5	17·8	13·4–24·0	10·1	8·14–13·0	11·8	9·06–16·7
*n*-3 fatty acids (g)||	0·42	0·28	0·53	0·29	0·57	0·32	0·27	0·16	0·27	0·15
*n*-6 fatty acids (g)¶	12·6	7·67–19·1	16·9	11·5–24·3	16·1	11·1–23·3	9·29	6·26–13·4	8·89	6·01–13·3
Onion (g)**	63·8	35·0–96·0	86·3	59·0–134	93·0	69·5–131	43·0	26·0–67·5	40·0	18·0–62·0
PUFA (g)	13·6	8·69–20·3	17·9	12·3–25·7	17·5	12·1–24·5	10·3	7·00–14·5	9·71	7·17–13·9
Riboflavin (mg)	1·29	0·80	1·54	0·84	1·66	0·79	0·86	0·51	1·14	0·75
Selenium (ug)	22·7	13·2–35·6	29·6	19·6–43·1	28·4	19·9–42·0	15·7	9·85–25·2	16·6	8·98–26·4
Thiamin (mg)	1·71	0·81	1·69	0·8	2·09	1·02	1·49	0·57	1·70	0·74
Vitamin A (RE)	636	362–1079	930	529–1501	882	554–1457	473	322–719	419	274–653
Vitamin B_6_ (mg)	1·52	0·80	1·67	0·88	1·95	0·95	1·20	0·46	1·29	0·56
Vitamin C (mg)	19·0	11·2–40·2	37·5	19·3–57·9	31·1	19·2–55·9	12·5	8·27–18·7	11·7	7·31–16·4
Vitamin D (μg)	2·18	1·12–3·69	2·8	1·56–4·46	3·12	1·85–4·76	1·56	0·84–2·56	1·62	0·89–2·51
Vitamin E (mg)	10·0	6·36–14·5	12·4	8·50–16·5	12·1	7·94–16·7	8·16	5·57–11·7	7·78	4·85–11·2
Zinc (mg)	10·3	4·89	11·2	4·82	13·3	6·04	8·05	2·92	9·29	3·86

PURE, Prospective Urban and Rural Epidemiology; NWP, North-West Province; SA, South Africa; AE-DII, adjusted energy-adjusted dietary inflammatory index; IQR, inter quartile range; RE, retinol.

Normally distributed variables are expressed as mean and sd. Not normally distributed variables are expressed as median and (25–75th percentiles).

* kJ (energy) is considered a pro-inflammatory component in the DII^®^ although AE-DII calculations (as performed in the current investigation) standardise food parameter intakes according to energy and do not include energy as a separate pro-inflammatory component.

† Alcohol intake > 40 g/d was treated as a missing value.

‡ Compiled by adding intake of both rooibos and black tea.

§ Caffeine 68 mg/100 ml coffee; caffeine 20 mg/100 ml tea, brewed.

|| *n*-3 fatty acids compiled by summing C18_3, C18_4, C20_5, C22_5 and C22_6.

¶ *n*-6 fatty acids compiled by summing C18_2 and C20_4.

** Compiled by adding individual intake of onion (boiled), onion (raw), onion (sautéed in sunflower oil), spinach (Swiss Chard, cooked with potato, onion and sunflower oil), Green beans (cooked with potato, onion and sunflower oil), carrot (cooked with potato, onion and sunflower oil), cabbage (cooked with potato, onion and sunflower oil), chicken (with skin, stew, tomato and onion), fish (casserole low-fat fish, tomato and onion sauce), tomato and onion (stewed no sugar), tomato and onion (stewed with sugar), tomato and onion (canned), beef (mince lean, savoury tomato and onion), beef (corned, savoury, potato and onion) and weighted according to the onion concentration in the recipe.

All of the seven pro-inflammatory food components differed across the four sex/rural–urban status groups (*P* < 0·001). Sex and rural–urban differences were observed in relation to carbohydrate, protein, cholesterol and Fe consumption (Mann–Whitney *U P*-values < 0·01 for all) while rural–urban status, rather than sex drove the aggregated demographic associations with saturated fat, trans fat and vitamin B_12_ (*P* < 0·001 for rural–urban comparison, *P* > 0·05 for sex comparisons).

With the exception of flavones and tea, all anti-inflammatory food and nutrient parameters also differed across sex/rural–urban status groups (*P* < 0·001). Rural–urban status drove these associations for SFA, MUFA and PUFA, selenium, vitamins A, B_12_, C and E, *n*-3 fatty acids, anthocyanidin, flavon-3-ol, flavonols and onion (rural–urban comparisons, *P* < 0·001 *v*. *P* > 0·05 for sex comparisons). Inversely, differences among isoflavones in the sex/rural–urban status demographic were driven by sex as opposed to rural–urban differences (*P* < 0·001 *v*. 0·14 in rural-urban *v*. sex comparisons, respectively). Out of the twenty-eight anti-inflammatory food components, only caffeine was consumed in higher quantity in rural *v*. urban participants, while twenty-five of the anti-inflammatory food components were consumed in higher quantities by urban participants and, in particular, the urban men (highest consumption of all four groups in 19/28 food parameters).

### The AE-DII in relation to inflammatory and cardio-metabolic risk markers

Table 3 summarises clinical markers of inflammation, cardio-metabolic risk and liver function across quartiles of the AE-DII scores in the PURE-NWP-SA cohort. Less than 1 % (*n* 12) and 27 % (*n* 507) of participants made intermittent use of anti-inflammatory medication and aspirin, respectively, at the time of data collection. The majority of the cohort was within the recommended healthy ranges for all risk markers, except for blood pressure (median systolic blood pressure/diastolic blood pressure: 130/87 mmHg).

**Table 3:** Clinical characteristics of the PURE-NWP-SA cohort overall and stratified by AE-DII quartiles

	Total cohort		AE-DII quartiles								
			1 (–3·708; –0·467)		2 (–0·466; 0·296)		3 (0·297; 1·174)		4 (1·175; 5·080)		*P*-value
	Mean/median	sd/IQR	Mean/median	sd/IQR	Mean/median	sd/IQR	Mean/median	sd/IQR	Mean/median	sd/IQR	
*n*	1858		465		465		464		464		
Inflammatory markers											
hs-CRP (mg/l)	3·31	1·04–9·35	3·90	1·02–11·7	3·17	0·88–8·61	3·05	1·06–8·65	3·25	1·11–9·49	0·71
IL-6 (pg/ml)	2·84	0·75–5·64	2·98	1·64–5·69	2·63	0·75–5·53	2·69	0·75–5·37	3·02	0·75–5·94	0·12
suPAR (ng/ml)	3·50	2·84–4·36	3·39	2·87–4·30	3·34*	2·76–4·20	3·65*	2·87–4·57	3·61	2·89–4·52	**0·02**
Albumin (g/dl)	3·51	2·59–6·83	3·57*^#^	2·67–6·50	3·50	2·55–7·26	3·61*	2·58–7·16	3·36^#^	2·56–6·51	**0·009**
Cardio-metabolic risk markers											
BMI (kg/m^2^)	23·1	19·4–29·0	23·5	19·6–29·8	23·5	19·5–28·8	23·0	19·0–29·0	22·2	19·1–27·7	0·51
Waist circumference (cm)	77·8	70·4–88·0	78·2	71·1–89·3	78·4	70·5–89·2	77·3	70·0–87·5	76·2	70·0–87·3	0·65
SBP (mmHg)	134	24·5	134	24·8	137	25·4	134	24·2	130	23·2	0·06
DBP (mmHg)	87·8	14·5	87·6	14·7	89·1	14·6	88·3	14·5	86·0	14·3	0·14
TC (mmol/l)	5·02	1·37	5·17	1·35	5·00	1·35	4·97	1·44	4·94	1·33	0·41
LDL-C (mmol/l)	2·91	1·19	3·03	1·17	2·87	1·22	2·9	1·17	2·84	1·17	0·59
HDL-C (mmol/l)	1·52	0·63	1·54	0·60	1·52	0·61	1·51	0·68	1·52	0·65	0·66
TGA (mmol/l)	1·08	0·82–1·55	1·09	0·85–1·60	1·14	0·83–1·61	1·04	0·78–1·49	1·07	0·80–1·50	0·21
Fasting glucose (mmol/l)	4·80	4·30–5·30	4·80	4·40–5·30	4·80	4·30–5·40	4·80	4·30–5·20	4·80	4·30–5·40	0·21
HbA1c (%)	5·60	5·30–5·80	5·60	5·30–5·90	5·60	5·23–5·80	5·50	5·20–5·80	5·50	5·20–5·80	0·90
ALT (μg/l)	17·0	12·0–25·0	16·7	12·6–23·7	16·4	11·8–23·0	16·91	11·6–25·6	19·0	13·0–26·7	**0·04**
AST (μg/l)	26·0	19·2–38·8	25·0*	19·0 –36·1	25·1^#^	19·0–4·00	25·7	19·3–38·2	28·7*^#^	20·0–44·9	**0·003**
GGT (μg/l)	46·1	30·0–88·0	44·0*	28·9–69·0	46·7^#^	31·1–85·4	43·9^&^	29·0–91·0	50·0*^#&^	31·1–119	**< 0·001**

PURE, Prospective Urban and Rural Epidemiology; NWP, North-West Province; SA, South Africa; AE-DII, adjusted energy-adjusted dietary inflammatory index; IQR, inter quartile range; hs-CRP, high sensitivity C-reactive protein; suPAR, soluble urokinase-type plasminogen activator receptor; SBP, systolic blood pressure; DBP, diastolic blood pressure; TC, total cholesterol; LDL-C, LDL cholesterol; HDL-C, HDL cholesterol; HbA1c, glycated Hb; ALT, alanine transaminase; AST, aspartate transaminase; GGT, gamma-glutamyl transferase.

*P*-values: ANCOVA adjusted for age, sex, dwelling status, education, smoking status, anti-inflammatory medication and aspirin use. Statistically significant associations are indicated in bold text.

Normally distributed variables are expressed as mean and sd. Not normally distributed variables are expressed as median (25–75th percentiles).

^*#&^Quartiles with the same symbol differ significantly from each other upon covariate adjustment as indicated by Tukey *post hoc* tests.

Albumin and soluble urokinase-type plasminogen activator receptor, but not CRP or IL-6 concentrations differed across AE-DII quartiles upon covariate adjustment. In the regression models, only serum albumin was associated with continuous AE-DII scores (0·94 g/l lower albumin per one-unit higher AE-DII; *P* < 0·001). This association remained robust upon additional adjustment for BMI, energy intake and alcohol consumption (–0·77 g/l per one-unit higher AE-DII, *P* = 0·004).

With the exception of the indicators of liver function (ALT, AST and GGT), no cardio-metabolic risk markers differed across AE-DII quartiles. When investigated on a continuous scale, inverse associations between the AE-DII and several cardio-metabolic risk markers (BMI, systolic blood pressure, diastolic blood pressure, total cholesterol and LDL-C) were observed only prior to covariate adjustment. In contrast, HDL-C is associated with the AE-DII only upon covariate adjustment in the secondary model. A one-unit higher AE-DII was associated with a 0·05 mmol/l lower HDL-C (*P* < 0·001). This association remained significant when serum albumin (the only inflammatory biomarker associated with the AE-DII upon full adjustment) was additionally adjusted for (regression coefficient: –0·03, *P* = 0·005). Similarly, associations between the AE-DII and fasting glucose and WC were only observed upon full covariate adjustment, including additional adjustment for serum albumin. Each one-unit higher AE-DII was associated with a 0·54 cm higher WC and a 0·07 mmol/L higher fasting glucose, (*P* = 0·036 and 0·034).

Increases of 2·4, 3·7 and 5·8 % were observed in ALT, AST and GGT concentrations, respectively, for a one-unit higher AE-DII (*P* < 0·01 for all, upon primary covariate adjustment). Associations were also robust to additional adjustment for BMI, although adjustment for daily alcohol consumption resulted in a complete loss of statistical significance (*P* > 0·05 for all). No associations between the AE-DII and TG or HbA_1c_ were observed.

## Discussion

This is the first study to describe and compare the dietary inflammatory potential of rural and urban Black South Africans following a typical African diet, including its demographic and lifestyle predictors and its association with various inflammatory and other cardio-metabolic risk markers.

### AE-DII in the PURE-NWP-SA cohort: the role of urbanicity

The dietary intakes reported by the PURE-NWP-SA cohort delivered AE-DIIs that spanned only 61 % of the theoretical range, emphasising the overall lack of diversity in dietary consumption. Dietary intakes differed between rural–urban status and sex. Urban participants consumed higher amounts of all nutrients with urban women consuming the most anti-inflammatory diet, followed by urban men. Rural participants had the most pro-inflammatory dietary pattern. Rural participants were furthermore less educated than urban participants, with lack of education itself being associated with a more pro-inflammatory diet. This is in agreement with Wirth *et al.* who report an inverse association between education and DII^®^ in African Americans^(26)^.

Although energy intake was relatively low overall, both the rural and urban participant groups reached dietary reference ranges for their respective carbohydrate and protein intakes, through a high consumption of maize, bread and sugar, which are the most affordable staple food products in these communities^(27)^. These macronutrients also contribute to the AE-DII as pro-inflammatory parameters together with saturated and trans fat. Rural participants consumed the highest proportion of carbohydrates while urban participants consumed the highest proportion of proteins. Although urban men consumed the highest amounts of four of the seven pro-inflammatory components, they also consumed the highest amounts of the majority of the anti-inflammatory components, resulting in the average AE-DII still being more anti-inflammatory than those of the rural participants. Conversely, rural men, who had on average the most pro-inflammatory diet, did not have the highest intakes of any of the pro-inflammatory components, suggesting that their intake of the anti-inflammatory components was proportionally even lower than the intakes in the other three groups.

The observation that the most anti-inflammatory AE-DII score observed reached only about 50 % of the theoretical maximum anti-inflammatory potential, highlights the low consumption of anti-inflammatory AE-DII food parameters in particular. It has previously been reported that the PURE-NWP-SA cohort consumed, on average, less than half the daily recommended fruit and vegetable intake^(13)^ which can, at least partly, account for the limited anti-inflammatory micronutrient representation. On average, participants in both the rural and urban groups reported consuming less than the recommended dietary reference intake for folate, magnesium, niacin, riboflavin, selenium, vitamin A, vitamin B_6_, vitamin C, vitamin D, vitamin E and zinc^(16)^. These nutrients constitute eleven of the twenty-eight anti-inflammatory AE-DII components and are abundant in fruit and vegetables. The lack of fruit and vegetable consumption is largely attributed to their high cost and limited availability, particularly in rural areas^(28)^.

### AE-DII in the PURE-NWP-SA cohort: the role of alcohol consumption

While alcohol intake is considered an anti-inflammatory component in the E-DII, this anti-inflammatory effect has been questioned for intakes in excess of 40 g/d^(23)^, supporting the exclusion of excessive alcohol consumers from previous DII^®^ investigations^(22)^. In agreement with this notion, in the PURE-NWP-SA population, the proportion of alcohol consumers as well as the amount of alcohol consumed increased across the AE-DII quartiles. Notably, the AE-DII scores investigated here were calculated without taking the alcohol consumption data of excessive drinkers into account (i.e. we omitted intake values > 40 g/d when calculating the AE-DII). The observation that the association between the AE-DII and alcohol intake was nullified when the 177 participants consuming more than 40 g of alcohol per day were removed from analysis, could suggest that the lifestyle associated with excessive drinking, e.g. smoking and level of education, rather than the ethanol content itself, predicts AE-DII scores. For example, the proportion of ever smokers dramatically increased across categories of alcohol consumption: non-drinkers (43 %), moderate drinkers (1–40 g/d, 71 %) and excessive drinkers (> 40 g/d, 79 %). A less dramatic, though similar pattern is also observed in terms of proportion of uneducated individuals (33 % *v*. 37 % *v*. 39 %). Lack of education and *ever* smoking status were both associated with adverse AE-DII scores in the PURE-NWP-SA. Similar observations of lower dietary quality in participants who smoke have previously been reported^(29,30)^.

While moderate alcohol consumption is often associated with a healthy lifestyle, such as observed in the Mediterranean diet, in this population it is clear that a number of participants consume excessive, rather than moderate, amounts of alcohol and that these individuals were also often smokers. These are more indicative of an unhealthy lifestyle which can contribute to a more pro-inflammatory diet and finally increase the likelihood of developing CVD.

It remains unclear to which degree the alcohol consumption data included in the DII^®^ development process relied upon the preferential consumption of flavonoid-rich sources such as wines and spirits typically associated with the Mediterranean diet^(31)^. Africans have, on the other hand, been reported to preferentially consume beer, including homebrewed rather than wine^(32)^. For this reason, the increased representation of African cohorts remains crucial as the seemingly contradictory observations presented here might emanate from the variable sources of ethanol preferred among and available to various communities. In addition, because of alcohol’s inclusion in the DII^®^ itself, limited data where alcohol consumption was explored beyond its incorporation in the calculated score exist. In the PURE-NWP-SA cohort, alcohol consumption was significantly associated with most AE-DII components upon adjustment for energy. Although alcohol consumption is negatively associated with pro-inflammatory AE-DII parameters and positively with numerous anti-inflammatory markers, it is also associated negatively with a number of anti-inflammatory AE-DII parameters including MUFA and PUFA, *n*-6 fatty acid, fibre and *β*-carotene (data not shown, adjusted *r* < –0·23, *P* < 0·001). Further research is warranted to determine whether different parameter-specific inflammatory effect scores should be allocated to moderate (anti-inflammatory) and excessive (pro-inflammatory) alcohol consumption.

### The AE-DII as a predictor of inflammation

Contrary to our hypothesis, apart from its association with albumin, AE-DII scores did not significantly predict inflammatory status in the PURE-NWP-SA cohort. The lack of association between the AE-DII and inflammatory markers in the PURE-NWP-SA cohort may be attributed to the limited variation in inflammatory potential represented by the AE-DII scores. Although the mean cohort AE-DII was indicative of a pro-inflammatory diet, it remains close to a null effect AE-DII (AE-DII≈0). Dietary consumption was therefore likely not sufficiently pro- or anti-inflammatory to modify serum inflammatory marker levels. While construct validation^(26,33)^ and other studies^(10,34–37)^ demonstrated an association between the DII^®^ and inflammatory markers, a number of studies also reported no association or found associations with only some but not all inflammatory markers^(26,33,38–40)^. Furthermore, several studies found no association between the DII^®^ and CRP or IL-6 as a continuous variable and only report associations for dichotomised outcomes (CRP > 3 mg/l or IL-6 > 1·6 pg/ml)^(8,33,40)^.

Low albumin concentrations have previously been used as indicators of low-grade systemic inflammation^(41)^. In the PURE-NWP-SA cohort, the negative AE-DII-albumin association could therefore be indicative of an association with low-grade chronic rather than acute inflammation. C-reactive protein, soluble urokinase-type plasminogen activator receptor and IL-6 concentrations are often used to characterise an acute inflammatory state^(42,43)^, explaining why, in the case of the PURE-NWP-SA cohort, these markers as opposed to albumin, did not significantly associate with the AE-DII. We have previously demonstrated that the PURE-NWP-SA cohort has high levels of acute inflammatory markers (e.g. a median CRP of 3·31 mg/l and IL-6 of 2·84 pg/ml, Table 3). Myburgh *et al.* identified a number of factors, other than diet, that were significantly associated with CRP specifically, such as age, socio-economic status, genetics and infectious diseases, thus potentially diluting the contribution of the inflammatory potential of the diet^(44)^.

### The AE-DII in relation to cardio-metabolic risk markers

The AE-DII was significantly associated with three of the cardio-metabolic risk markers investigated in this study: HDL-C, fasting glucose and WC. These associations were in line with prior findings^(35,40)^ and seemed to be mediated by inflammation (represented by albumin) as the associations were strengthened (HDL-C) or became significant (fasting glucose and WC) upon adjustment for albumin.

Insulin resistance has been suggested as the main mechanism behind the association of E-DII with glucose as low-grade chronic inflammation associates with the intracellular signalling pathways that are involved in the regulation of insulin^(45)^. Inhibition of these signalling pathways through inflammation results in insulin resistance^(46)^, which could be the cause of increased fasting glucose concentrations. Furthermore, increased fasting blood glucose concentrations may be attributed to low dietary fibre intake. The mean fibre intake for this cohort was 21·7 g which is below the Dietary Reference Intake for both men (38 g) and women (25 g)^(47)^. Dietary fibre delays the absorption of glucose which consequently reduces fasting blood glucose and insulin levels^(48)^.

In terms of markers of liver function, positive associations between the AE-DII, GGT, ALT and AST were observed. As opposed to prior observations^(49,50)^, the AE-DII was not associated with increased GGT, AST and ALT levels in the PURE-NWP-SA population through the pathway of obesity/fatty liver disease, as the associations remained after adjustment. Instead the associations appear, at least in part, to be related to alcohol consumption, as adjustment therefore nullified the association between the AE-DII and the liver enzymes. Alcohol consumption is known to affect liver enzyme concentrations, with GGT being the most susceptible to its effects^(51)^. GGT increases with alcohol intake^(52)^ and can be used to verify reported alcohol intake^(53–55)^. These findings suggest that, while the final calculated inflammatory index of the diet is positively associated with liver enzymes, the factors contributing to the final AE-DII score of a participant in this population probably differ from those in more affluent Western populations. These differences are likely anchored in the overall low intakes of food components and total energy as reflected by intakes of a number of components being lower than the recommended ranges, as well as the monotonous nature of the diet consumed and the observed high use of alcohol and tobacco.

### Limitations

Although this study did not set out to investigate disease incidence or causality, the cross-sectional nature of the data limited any option to do so. The limited variety in the PURE-NWP-SA participants’ diet as well as the monotonous dietary pattern observed in this study population can contribute to poor associations between the AE-DII and inflammation. This is not a limitation of this study *per se*, but rather highlights the lack of availability of population-specific dietary indicators. In the same vein, the available computational tools for flavonoid quantification do not allow for the inclusion of rooibos tea rich in flavonols and flavan-3-ol, which is the most frequently consumed tea in this cohort and in South Africa. We were however, able to partially account for rooibos tea’s beneficial properties by including it in the *tea* AE-DII component. In addition, we used our own population’s mean and sd dietary intake rather than the energy-adjusted global comparative database to standardise intakes. This prohibits direct comparison with published E-DII data. Finally, we were unable to investigate alcohol consumption patterns in this study due to the nature of a recall dietary questionnaire that reports alcohol consumption as average intake per day. Taking patterns such as binge drinking into account could have further elucidated the role of alcohol consumption in the AE-DII.

## Conclusion

This study described, for the first time, the inflammatory potential of a typical South African diet consumed by continental Africans residing in distinct rural and urban sites. Overall, more pro-inflammatory food components and nutrients were consumed in proportionally higher quantities than anti-inflammatory components, resulting in a pro-inflammatory average AE-DII in the PURE-NWP-SA cohort. Urban women followed the most anti-inflammatory diet and rural men the most pro-inflammatory diet. The diet of the participants was not overtly pro- or anti-inflammatory and therefore had little effect on the inflammatory markers. Our data also demonstrate that the inflammatory potential of alcohol at different levels of intake requires further research. The results of this study highlight the need to better understand the role of the inflammatory potential of the diet in the context of food insecurity, unhealthy lifestyle practices and increased risk for NCD, all of which are highly prevalent in developing countries such as South Africa.

## Supplementary material

For supplementary material accompanying this paper visit https://doi.org/10.1017/S136898002100505X. click here to view supplementary material

## References

[1] Stanaway JD , Afshin A , Gakidou E et al. (2018) Global, regional, and national comparative risk assessment of 84 behavioural, environmental and occupational, and metabolic risks or clusters of risks for 195 countries and territories, 1990–2017: a systematic analysis for the Global Burden of Disease Study 2017. Lancet 392, 1923–1994. 30496105 10.1016/S0140-6736(18)32225-6 PMC6227755

[2] Bennett JM , Reeves G , Billman GE et al. (2018) Inflammation–nature’s way to efficiently respond to all types of challenges: implications for understanding and managing ‘the epidemic’ of chronic diseases. Front Med 5, 316. 10.3389/fmed.2018.00316 PMC6277637 30538987

[3] Koch W (2019) Dietary polyphenols – important non-nutrients in the prevention of chronic noncommunicable diseases. A systematic review. Nutrients 11, 1039. 31075905 10.3390/nu11051039 PMC6566812

[4] Proctor MJ , McMillan DC , Horgan PG et al. (2015) Systemic inflammation predicts all-cause mortality: a glasgow inflammation outcome study. PLOS ONE 10, e0116206. 25730322 10.1371/journal.pone.0116206 PMC4346265

[5] Dinu M , Pagliai G , Casini A et al. (2018) Mediterranean diet and multiple health outcomes: an umbrella review of meta-analyses of observational studies and randomised trials. Eur J Clin Nutr 72, 30–43. 28488692 10.1038/ejcn.2017.58

[6] Ricker MA & Haas WC (2017) Anti-inflammatory diet in clinical practice: a review. Nutr Clin Pract 32, 318–325. 28350517 10.1177/0884533617700353

[7] Christ A , Lauterbach M & Latz E (2019) Western diet and the immune system: an inflammatory connection. Immunity 51, 794–811. 31747581 10.1016/j.immuni.2019.09.020

[8] Cavicchia PP , Steck SE , Hurley TG et al. (2009) A new dietary inflammatory index predicts interval changes in serum high-sensitivity C-reactive protein. J Nutr 139, 2365–2372. 19864399 10.3945/jn.109.114025 PMC2777480

[9] Shivappa N , Steck SE , Hurley TG et al. (2014) Designing and developing a literature-derived, population-based dietary inflammatory index. Public Health Nutr 17, 1689–1696. 23941862 10.1017/S1368980013002115 PMC3925198

[10] Shivappa N , Wirth MD , Hurley TG et al. (2017) Association between the dietary inflammatory index (DII) and telomere length and C-reactive protein from the National Health and Nutrition Examination Survey-1999–2002. Mol Nutr Food Res 61, 1600630. 10.1002/mnfr.201600630 PMC5380547 27878970

[11] Marx W , Veronese N , Kelly JT et al. (2021) The dietary inflammatory index and human health: an umbrella review of meta-analyses of observational studies. Adv Nutr 12, 1681–1690. 33873204 10.1093/advances/nmab037 PMC8483957

[12] Teo K , Chow CK , Vaz M et al. (2009) The Prospective Urban Rural Epidemiology (PURE) study: examining the impact of societal influences on chronic noncommunicable diseases in low-, middle-, and high-income countries. Am Heart J 158, 1–7. 19540385 10.1016/j.ahj.2009.04.019

[13] Dolman RC , Wentzel-Viljoen E , Jerling JC et al. (2014) The use of predefined diet quality scores in the context of CVD risk during urbanization in the South African Prospective Urban and Rural Epidemiological (PURE) study. Public Health Nutr 17, 1706–1716. 23952977 10.1017/S1368980013002206 PMC10282358

[14] Mchiza ZJ , Steyn NP , Hill J et al. (2015) A review of dietary surveys in the adult South African population from 2000 to 2015. Nutrients 7, 8227–8250. 26404371 10.3390/nu7095389 PMC4586583

[15] World Health Organization (2018) World Health Statistics 2018: Monitoring Health for the SDGs, Sustainable Development Goals. Geneva: WHO.

[16] Wentzel-Viljoen E , Lee S , Laubscher R et al. (2018) Accelerated nutrition transition in the North West Province of South Africa: results from the Prospective Urban and Rural Epidemiology (PURE-NWP-SA) cohort study, 2005–2010. Public Health Nutr 21, 2630–2641. 29734966 10.1017/S1368980018001118 PMC10260886

[17] Vorster HH , Kruger A , Wentzel-Viljoen E et al. (2014) Added sugar intake in South Africa: findings from the adult prospective urban and rural epidemiology cohort study. Am J Clin Nutr 99, 1479–1486. 24740206 10.3945/ajcn.113.069005

[18] MacIntyre U , Venter C & Vorster H (2001) A culture-sensitive quantitative food frequency questionnaire used in an African population: 2. Relative validation by 7-day weighed records and biomarkers. Public Health Nutr 4, 63–71. 11315682 10.1079/phn200041

[19] Venter CS , MacIntyre UE & Vorster HH (2000) The development and testing of a food portion photograph book for use in an African population. J Hum Nutr Diet 13, 205–218. 12383127 10.1046/j.1365-277x.2000.00228.x

[20] Wolmarans P , Danster N , Dalton A et al. (2010) Condensed Food Composition Tables for South Africa. Parow Valley, Cape Town: Medical Research Council.

[21] Langenhoven M , Conradie P , Wolmarans P et al. (1991) MRC Food Quantities Manual, 2nd ed. Parow Valley, Cape Town: Medical Research Council.

[22] van Woudenbergh GJ , Theofylaktopoulou D , Kuijsten A et al. (2013) Adapted dietary inflammatory index and its association with a summary score for low-grade inflammation and markers of glucose metabolism: the Cohort study on Diabetes and Atherosclerosis Maastricht (CODAM) and the Hoorn study. Am J Clin Nutr 98, 1533–1542. 24153342 10.3945/ajcn.112.056333

[23] Imhof A , Woodward M , Doering A et al. (2004) Overall alcohol intake, beer, wine, and systemic markers of inflammation in western Europe: results from three MONICA samples (Augsburg, Glasgow, Lille). Eur Heart J 25, 2092–2100. 15571824 10.1016/j.ehj.2004.09.032

[24] Roberts WC (1988) The Friedewald-Levy-Fredrickson formula for calculating low-density lipoprotein cholesterol, the basis for lipid-lowering therapy. Am J Cardiol 62, 345–346. 3165237 10.1016/0002-9149(88)90248-2

[25] R Core Team (2020) R: A Language and Environment for Statistical Computing. Vienna, Austria: R Foundation for Statistical Computing.

[26] Wirth MD , Shivappa N , Davis L et al. (2017) Construct validation of the dietary inflammatory index among African Americans. J Nutr Health Aging 21, 487–491. 28448077 10.1007/s12603-016-0775-1 PMC5547883

[27] Ronquest-Ross L-C , Vink N & Sigge GO (2015) Food consumption changes in South Africa since 1994. S Afr J Sci 111, 1–12.

[28] Wentzel-Viljoen E , Laubscher R & Vorster HH (2018) Changes in food intake from 2005 to 2010 by a cohort of black rural and urban African men and women in the North West Province of South Africa: the PURE-NWP-SA study. Public Health Nutr 21, 2941–2958. 30149823 10.1017/S1368980018001878 PMC10260798

[29] Alkerwi Aa , Baydarlioglu B , Sauvageot N et al. (2017) Smoking status is inversely associated with overall diet quality: findings from the ORISCAV-LUX study. Clin Nutr 36, 1275–1282. 27595637 10.1016/j.clnu.2016.08.013

[30] Maisonneuve P , Shivappa N , Hébert JR et al. (2016) Dietary inflammatory index and risk of lung cancer and other respiratory conditions among heavy smokers in the COSMOS screening study. Eur J Nutr 55, 1069–1079. 25953452 10.1007/s00394-015-0920-3 PMC4846387

[31] Hodge A , Bassett J , Shivappa N et al. (2016) Dietary inflammatory index, Mediterranean diet score, and lung cancer: a prospective study. Cancer Causes Control 27, 907–917. 27294725 10.1007/s10552-016-0770-1 PMC5550291

[32] Trangenstein PJ , Morojele NK , Lombard C et al. (2018) Heavy drinking and contextual risk factors among adults in South Africa: findings from the International Alcohol Control study. Subst Abuse Treat Prev Policy 13, 1–11. 30518429 10.1186/s13011-018-0182-1 PMC6280515

[33] Tabung FK , Steck SE , Zhang J et al. (2015) Construct validation of the dietary inflammatory index among postmenopausal women. Ann Epidemiol 25, 398–405. 25900255 10.1016/j.annepidem.2015.03.009 PMC4433562

[34] Wirth MD , Sevoyan M , Hofseth L et al. (2018) The Dietary Inflammatory Index is associated with elevated white blood cell counts in the National Health and Nutrition Examination Survey. Brain Behav Iimmun 69, 296–303. 10.1016/j.bbi.2017.12.003 PMC5857420 29217263

[35] Phillips C , Shivappa N , Hébert J et al. (2018) Dietary inflammatory index and biomarkers of lipoprotein metabolism, inflammation and glucose homeostasis in adults. Nutrients 10, 1033. 30096775 10.3390/nu10081033 PMC6115860

[36] Shivappa N , Steck SE , Hurley TG et al. (2014) A population-based dietary inflammatory index predicts levels of C-reactive protein in the Seasonal Variation of Blood Cholesterol Study (SEASONS). Public Health Nutr 17, 1825–1833. 24107546 10.1017/S1368980013002565 PMC3983179

[37] Shivappa N , Wirth MD , Murphy EA et al. (2019) Association between the Dietary Inflammatory Index (DII) and urinary enterolignans and C-reactive protein from the National Health and Nutrition Examination Survey-2003–2008. Eur J Nutr 58, 797–805. 29675557 10.1007/s00394-018-1690-5

[38] Shivappa N , Hebert JR , Marcos A et al. (2017) Association between dietary inflammatory index and inflammatory markers in the HELENA study. Mol Nutr Food Res 61, 1600707. 10.1002/mnfr.201600707 PMC5517083 27981781

[39] Shivappa N , Hébert JR , Rietzschel ER et al. (2015) Associations between dietary inflammatory index and inflammatory markers in the Asklepios Study. Br J Nutr 113, 665–671. 25639781 10.1017/S000711451400395X PMC4355619

[40] Wirth M , Burch J , Shivappa N et al. (2014) Association of a dietary inflammatory index with inflammatory indices and the metabolic syndrome among police officers. J Occup Environ 56, 986. 10.1097/JOM.0000000000000213 PMC4156884 25046320

[41] Soeters PB , Wolfe RR & Shenkin A (2019) Hypoalbuminemia: pathogenesis and clinical significance. J Parenter Enter Nutr 43, 181–193. 10.1002/jpen.1451 PMC7379941 30288759

[42] Koch A , Voigt S , Kruschinski C et al. (2011) Circulating soluble urokinase plasminogen activator receptor is stably elevated during the first week of treatment in the intensive care unit and predicts mortality in critically ill patients. Crit Care 15, 1–14. 10.1186/cc10037 PMC3221996 21324198

[43] Gabay C & Kushner I (1999) Acute-phase proteins and other systemic responses to inflammation. NEJM 340, 448–454. 9971870 10.1056/NEJM199902113400607

[44] Myburgh PH (2018) Interactions of CRP-SNPs with Selected Contributing Factors in Determining CRP Concentrations in Black South Africans. PhD Thesis. North-West University. https://repository.nwu.ac.za/handle/10394/31558 (accessed December 2021).

[45] White MF (1997) The insulin signalling system and the IRS proteins. Diabetologia 40, S2–S17. 9248696 10.1007/s001250051387

[46] Hotamisligil GS , Peraldi P , Budavari A et al. (1996) IRS-1-mediated inhibition of insulin receptor tyrosine kinase activity in TNF-*α*-and obesity-induced insulin resistance. Science 271, 665–670. 8571133 10.1126/science.271.5249.665

[47] Escott-Stump S & Mahan L (2008) Dietary Reference Intakes (DRIs): Recommended Intakes for Individuals, Vitamin/Mineral. Krause’s Food & Nutrition Therapy, 12th ed. Philadelphia: Saunders Elsevier.

[48] McRae MP (2018) Dietary fiber intake and type 2 diabetes mellitus: an umbrella review of meta-analyses. J Chiropr Med 17, 44–53. 29628808 10.1016/j.jcm.2017.11.002 PMC5883628

[49] Darbandi M , Hamzeh B , Ayenepour A et al. (2020) Anti-inflammatory diet consumption reduced fatty liver indices: finding from a large cohort study in the Kurdish population. Sci Rep 11, 22601. 10.1038/s41598-021-98685-3 PMC8604894 34799655

[50] Vahid F , Shivappa N , Hekmatdoost A et al. (2019) Association of pro-inflammatory dietary intake and non-alcoholic fatty liver disease: findings from Iranian case-control study. Int J Vitam Nutr Res 88, 144–150. 10.1024/0300-9831/a000571 30887902

[51] Salaspuro M (1999) Carbohydrate-deficient transferrin as compared to other markers of alcoholism: a systematic review. Alcohol 19, 261–271. 10580517 10.1016/s0741-8329(99)00044-0

[52] Teschke R & Koch T (1986) Biliary excretion of gamma-glutamyltransferase: selective enhancement by acute ethanol administration. Biochem Pharmacol 35, 2521–2525. 2874809 10.1016/0006-2952(86)90049-3

[53] Anttila P , Järvi K , Latvala J et al. (2003) Diagnostic characteristics of different carbohydrate-deficient transferrin methods in the detection of problem drinking: effects of liver disease and alcohol consumption. Alcohol Alcohol 38, 415–420. 12915516 10.1093/alcalc/agg102

[54] Jeppsson J , Kristensson H & Fimiani C (1993) Carbohydrate-deficient transferrin quantified by HPLC to determine heavy consumption of alcohol. Clin Chem 39, 2115–2120. 8403395

[55] Keating J , Cheung C , Peters TJ et al. (1998) Carbohydrate deficient transferrin in the assessment of alcohol misuse: absolute or relative measurements? A comparison of two methods with regard to total transferrin concentration. Clin Chim Acta 272, 159–169. 9641357 10.1016/s0009-8981(98)00008-4

