# Supplementary material for: The association between an energy-adjusted dietary inflammatory index and inflammation in rural and urban Black South Africans
Source: Public Health Nutr. 2021 Dec 27;25(12):3432–44. doi: 10.1017/S136898002100505X (PMC9991709; doi:10.1017/S136898002100505X)
Supplement: Supplementary file 1 [file S136898002100505Xsup.zip › S136898002100505Xsup001.docx]

Supplementary Table 1: Nutrients and food components included in PURE-NWP-SA 2005 data

| **Food or nutrient** | **Present in PURE 2005**  **Yes/No** |
| --- | --- |
| **Pro-inflammatory** | |
| Carbohydrate (g) | Yes |
| Cholesterol (mg) | Yes |
| Energy (kcal) | Yes |
| Iron (mg) | Yes |
| Protein (g) | Yes |
| Saturated fat (g) | Yes |
| Trans fat (g) | Yes |
| Vitamin B12 (µg) | Yes |
| **Anti-inflammatory** | |
| Alcohol (g) | Yes |
| Anthocyanidins (mg) | Yes |
| Beta-carotene (µg) | Yes |
| ^§^ Black tea/ Rooibos (g) | Yes |
| ‘*Caffeine (g) | Yes |
| Eugenol (mg) | No |
| Fibre (g) | Yes |
| Flavan-3-ol (mg) | Yes |
| Flavones (mg) | Yes |
| Flavonols (mg) | Yes |
| Flavonones (mg) | Yes |
| Folic acid (µg) | Yes |
| Garlic (g) | Not reported |
| Ginger (g) | Not reported |
| Isoflavones (mg) | Yes |
| Mono-unsaturated fatty acids (g) | Yes |
| Magnesium (mg) | Yes |
| Niacin (mg) | Yes |
| ° omega-3 fatty acids (g) | Yes |
| ᴛ omega-6 fatty acids (g) | Yes |
| ^ᴥ^ Onion (g) | Yes |
| Pepper (g) | No |
| Poly-unsaturated fatty acids (g) | Yes |
| Riboflavin (mg) | Yes |
| Rosemary (mg) | No |
| Saffron (g) | No |
| Selenium (µg) | Yes |
| Thiamin (mg) | Yes |
| Thyme/oregano (mg) | No |
| Total fat (g) | Yes |
| Turmeric (mg) | No |
| Vitamin A (RE) | Yes |
| Vitamin B6 (mg) | Yes |
| Vitamin C (mg) | Yes |
| Vitamin D (µg) | Yes |
| Vitamin E (mg) | Yes |
| Zinc (mg) | Yes |
| **Score range:**  -7.65 (anti-inflammatory) to 6.67 (pro-inflammatory) | |

‘* caffeine 68mg / 100ml coffee; caffeine 20mg / 100ml tea, brewed

° omega-3 fatty acids compiled by summing C18_3, C18_4, C20_5, C22_5 and C22_6

ᴛ omega-6 fatty acids compiled by summing C18_2 and C20_4

ᴥ compiled by adding individual intake of onion (boiled), onion (raw), onion (sautéed in sunflower oil), spinach (Swiss Chard, cooked with potato, onion, sunflower oil), Green beans (cooked with potato, onion and sunflower oil), carrot (cooked with potato, onion and sunflower oil), cabbage (cooked with potato, onion and sunflower oil), chicken (with skin, stew, tomato and onion), fish (casserole low-fat fish, tomato and onion sauce), tomato and onion (stewed no sugar), tomato and onion (stewed with sugar), tomato and onion (canned), beef (mince lean, savoury tomato, onion), beef (corned, savoury, potato, onion) and weighted according to the onion concentration in the recipe.

§ compiled by adding intake of both rooibos and black tea

**Supplementary table 2:** Comparison of the study variables between men and women with rural or urban dwelling status

|  | | **Urban** | | | | | **Rural** | | | |
| --- | --- | --- | --- | --- | --- | --- | --- | --- | --- | --- |
|  | | **Women** | | **Men** | | **Women** | | | **Men** | |
|  | | **Mean/ Median / % ^&^** | **SD /IQR** | **Mean/ Median / % ^&^** | **SD /IQR** | **Mean/ Median / % ^&^** | | **SD / IQR** | **Mean/ Median / % ^&^** | **SD / IQR** |
| N | | 578 |  | 390 |  | 585 | |  | 305 |  |
| **Demographics and lifestyle** | |  |  |  |  |  | |  |  |  |
| Age (y) | | 49.0 () | (43.0; 59.0) | 49.0 | (42.0; 58.0) | 46.9 | | (41.0; 54.0) | 49.0 | (42.0; 57.0) |
| Education (%) | |  |  |  |  |  | |  |  |  |
|  | *None* | 20.0 |  | 22.8 |  | 48.6 | |  | 52.8 |  |
|  | *1-12 years* | 78.9 |  | 75.6 |  | 51.3 | |  | 45.8 |  |
|  | *>12 years* | 1.10 |  | 1.61 |  | 0.18 | |  | 1.4 |  |
| Energy intake (kJ/day) | | 8700 | (6269; 115280 | 9464 | (7212; 12386) | 6021 | | (4925; 7523) | 6786 | (5413; 8594) |
| Alcohol consumption | |  |  |  |  |  | |  |  |  |
|  | *Status (% ever)* | 42.0 |  | 74.0 |  | 20.0 | |  | 56.0 |  |
|  | *Per day (g)* | 0.00 | (0.00; 11.5) | 11.6 | (0.00; 26.7) | 0.00 | | (0.00; 0.00) | 3.86 | (0.00; 28.9) |
| Smoker (% ever) | | 48.3 |  | 71.1 |  | 51.2 | |  | 62.1 |  |
| **Inflammatory markers** | |  |  |  |  |  | |  |  |  |
| hsCRP (mg/L) | | 4.19 | (1.48; 12.2) | 2.36 | (0.85; 7.55) | 3.51 | | (1.16; 9.30) | 3.03 | (0.71; 8.18) |
| IL-6 (pg/mL) | | 3.21 | (1.87; 5.75) | 2.72 | (0.75; 5.05) | 2.40 | | (0.75; 5.16) | 2.92 | (0.75; 7.15) |
| suPAR (ng/mL) | | 3.57 | (2.96; 4.52) | 3.40 | (2.73; 4.33) | 3.47 | | (2.85; 4.21) | 3.62 | (2.77; 4.50) |
| Albumin (g/L) | | 43.3 | (38.8; 58.0) | 43.0 | (37.0; 57.1) | 43.5 | | (40.3; 57.3) | 43.6 | (40.6; 56.3) |
| **Cardio-metabolic risk markers** | |  |  |  |  |  | |  |  |  |
| BMI (kg/m^2^) | | 27.6 | (22.5; 32.8) | 19.9 | (18.2; 22.8) | 24.9 | | (20.9; 30.7) | 19.7 | (18.0; 22.2) |
| Waist circumference (cm) | | 83.2 | (73.2; 92.7) | 74.4 | (69.7; 81.8) | 79.0 | | (69.6; 89.5) | 74.6 | (70.1; 80.5) |
| SBP (mmHg) | | 137 | 25.5 | 139 | 24.0 | 128 | | 22.7 | 132 | 24.4 |
| DBP (mmHg) | | 90.1 | 14.2 | 88.5 | 14.8 | 86.6 | | 14.1 | 84.6 | 15.0 |
| TC (mmol/L) | | 5.20 | 1.4 | 4.90 | 1.34 | 5.09 | | 1.37 | 4.71 | 1.31 |
| LDL-C (mmol/L) | | 3.06 | 1.19 | 2.70 | 1.19 | 3.04 | | 1.17 | 2.65 | 1.13 |
| HDL-C (mmol/L) | | 1.47 | 0.63 | 1.61 | 0.67 | 1.50 | | 0.6 | 1.54 | 0.65 |
| TG (mmol/L) | | 1.19 | (0.89;1.78) | 1.00 | (0.79; 1.47) | 1.09 | | (0.81;1.47) | 0.96 | (0.76; 1.34) |
| Glucose (mmol/L) | | 5.00 | (4.30; 5.50) | 4.80 | (4.20; 5.40) | 4.80 | | (4.40; 5.20) | 4.70 | (4.30; 5.10) |
| HbA1c (%) | | 5.60 | (5.30; 6.00) | 5.50 | (5.10; 5.70) | 5.60 | | (5.30; 5.82) | 5.50 | (5.20; 5.70) |
| **Liver function** | |  |  |  |  |  | |  |  |  |
| ALT (IU/L) | | 16.0 | (11.7; 23.8) | 19.3 | (13.0; 29.5) | 15.8 | | (11.6; 21.5) | 19.6 | (14.0; 29.5) |
| AST (IU/L) | | 24.1 | (19.0; 34.0) | 31.0 | (23.7; 49.5) | 23.8 | | (17.5; 34.0) | 28.6 | (20.0; 45.4) |
| GGT (IU/L) | | 43.3 | (28.6; 83.6) | 64.0 | (39.0; 132) | 37.8 | | (25.7; 60.2) | 52.0 | (33.8; 107) |

^&^ Percentage of individuals in each category

hs-CRP, high-sensitivity C-reactive protein. IL-6, interleukin 6. suPAR, soluble urokinase-type plasminogen activator receptor. DBP, diastolic blood pressure. SBP, systolic blood pressure. TC, total cholesterol., HDL-C, High-density lipoprotein cholesterol. LDL-C, Low-density lipoprotein cholesterol. TG, triglyceride. HbA1c, glycated haemoglobin. BMI, body mass index. ALT, Alanine transaminase. AST, Aspartate transaminase. GGT, Gamma-glutamyl transferase. SD, standard deviation. IQR, interquartile range.

Normally distributed variables are expressed as mean value and standard deviation

Not normally distributed variables are expressed as median (25–75th percentiles)
